# Rapid and Simple Determination of Sarafloxacin and Difloxacin in Beef by Capillary Electrophoresis Coupled with Solid-Phase Extraction

**DOI:** 10.1155/2018/1981436

**Published:** 2018-02-20

**Authors:** Qian Wang, Rui Zhao, Yan Cui, Cuicui Liu, Liya Liang, Shijie Yan

**Affiliations:** ^1^Department of Animal Science and Veterinary Medicine, Tianjin Agricultural University, Tianjin 300384, China; ^2^College of Food Science and Engineering, Northwest A&F University, Yangling, Shaanxi 712100, China; ^3^Department of Food Science and Biological Engineering, Tianjin Agricultural University, Tianjin 300384, China; ^4^Tianjin Engineering and Technology Research Center of Agricultural Products Processing, Tianjin 300384, China

## Abstract

A simple and rapid capillary electrophoresis method with diode array detector was developed for determination of sarafloxacin and difloxacin in beef. In this study, the experimental parameters affecting the determination were systematically optimized, including wavelength, buffer system, pH and concentration, and separation temperature and voltage. Under the optimal conditions, sarafloxacin and difloxacin could be quantified within 4 min using H_3_BO_3_/Na_2_B_4_O_7_ buffer (35 mmol/L, pH 8.8) as background electrolyte, 25 kV as separation voltage, and 22°C as the column temperature. The linear range of the method was 1–20 *μ*g/mL with LOD 0.8 *μ*g/mL for sarafloxacin and 0.5–20 *μ*g/mL with LOD 0.3 *μ*g/mL for difloxacin. The RSDs for the peak area of 8 *μ*g/mL sarafloxacin were 4.8% (intraday) and 7.8% (interday), respectively. The proposed method has been applied to determine the residue of sarafloxacin and difloxacin in beef samples with the satisfactory recovery.

## 1. Introduction

Fluoroquinolone (FQ) is the most important group of broad-spectrum antibiotics and are widely used in clinical practice for human and veterinary to treat and prevent various bacterial infections [1]. Difloxacin (DIF) and its metabolite sarafloxacin (SAR) belong to FQ antibacterials and usually work by inhibition of bacterial DNA gyrase to inhibit cell reproduction [2, 3]. Now, they are extensively used for prophylaxis and the treatment of diseases and as feed additives for mass gain promotion. However, the widespread usage of these antibacterials may bring about other potential negative effects. For example, the misuse of these antibacterials may lead to their presence in foodstuffs of animal origin and consequently increase the resistant human pathogens constituting a public health hazard [4, 5]. In the European Union (EU), the presence of these drugs in foodstuffs has been regulated through the Commission Regulation (EU) number 37/2010, and maximum residue limits (MRLs) have been established for different food matrices of animal origin at community level under Council Regulation number 470/2009. The maximum residue limit (MRL) for DIF is 400 *μ*g/kg in muscle of cattle, and the MRLs in other biological materials are between 100 and 1400 *μ*g/kg. MRLs of 30 mg/kg are recommended for SAR in fish muscle, 100 mg/kg in chicken liver, and 10 mg/kg in skin and fat; however, there is no regulation, at the moment, for the SAR in beef. Therefore, it is important to develop accurate and effective method for detecting SAR and DIF.

Recently, the methods for FQ residue analysis in food matrices of animal origin were mainly based on high-performance liquid chromatography with fluorometry or mass spectrometry and immunoassay [6–15]. Most of them suffer from tedious procedures, high solvent, and sample consumption. As a modern separation technique, capillary electrophoresis (CE) is very promising. It can be applied to food analysis because of its advantages, such as higher separation efficiency, high analysis speed, and very small consumption of expensive reagents and toxic solvents [16, 17]. Solid-phase extraction (SPE), the most common and well-established sample pretreatment technique, is usually used for the cleanup of complex matrices and preconcentration of target analytes [18]. CE coupled with SPE technology has been validated with satisfying results via the determination of DIF and SAR in chicken muscle [2]. Thus, we have reasons to expect that CE-SPE will continue its contribution to detection of other foodstuffs.

In this paper, we established an effective and reliable method allowing the determination of DIF and its metabolite SAR in beef by CE-SPE. The effects of buffer system, pH and concentration, and separation temperature and voltage on the separation and determination of SAR and DIF were systematically studied. The present method was further applied for the determination of SAR and DIF in beef. The result indicated that the method possessed the potential for rapidly and simply detecting SAR and DIF residues in food samples.

## 2. Materials and Methods

### 2.1. Materials and Chemicals

Acetonitrile, methanol, dichloromethane, and hexane (HPLC grade) were provided by Fisher Company. SAR hydrochloride (99.6%) and DIF (99.9%) were obtained from China Institute of Veterinary Drugs Control. C_18_ Solid-phase extractions were purchased from Bonna-Agela Technologies. Sodium hydroxide (NaOH), boric acid, and borax (analytical grade) were purchased from Thermo Fisher Scientific. Doubly deionized water (DDW, 18 MΩ cm) was prepared by a Milli-Q system (Millipore Corporation, Billerica, MA, USA).

H_3_BO_3_-Na_2_B_4_O_7_ buffer was prepared by mixing 35 mmol/L H_3_BO_3_ solution with 35 mmol/L Na_2_B_4_O_7_ solution to the required pH. All the buffer solutions were made with DDW, filtered through 0.22 *μ*m membrane filters, and degassed in an ultrasonic bath for 1 min before use.

### 2.2. Instrumentation

FLUKO FA25 high speed homogenizer (Darmstadt, Germany) was used to homogenize the beef sample. A high-speed refrigerated centrifuge (Thermo Fisher, Waltham, MA, USA) was used for centrifugation. The pH of the buffer was measured with a PB-10 potentiometer (Mettler Toledo, Zurich, Switzerland). CE analyses were run on a P/AGE system (Beckman Instruments, Palo Alto, CA, USA) with a diode array detector. Beckman 32.0 Kraft software was used for system control, data collection, and electropherogram integration. An untreated fused-silica capillary with an inner diameter of 50 *μ*m and total length of 75 cm (65 cm to the detector) was purchased from the Yongnian Optic Fiber Plant (Hebei, China). A vacuum rotary evaporator (Rongsheng, Minhang District, Shanghai, China) was used for concentration of the extracts.

New capillaries were successively pretreated with methanol, H_2_O, 0.1 mol/L NaOH, H_2_O, 0.1 mol/L HCl, and H_2_O for 5, 5, 30, 5, 30, and 5 min, respectively. Each day before the measurements, the capillary was conditioned with 0.1 mol/L NaOH and H_2_O for 10 min, followed by rinsing with the running buffer for 10 min. In order to keep the capillary wall in good condition, 0.1 mol/L NaOH filled it when it was not being used.

### 2.3. Spiking Control Sample and Extraction Procedure

**Figure 1:**
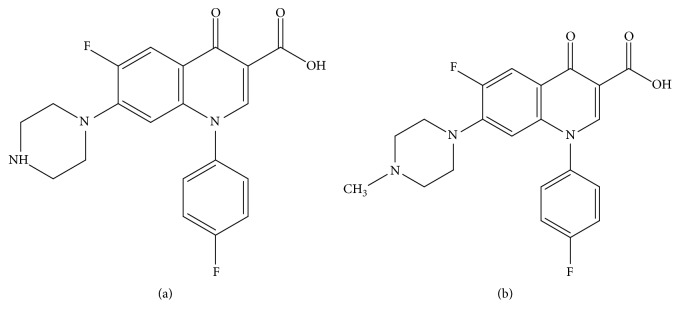
The chemical structure of (a) sarafloxacin and (b) difloxacin.

SAR and DIF are usually present in food matrices of animal origin (Chemical structures of the FQ are shown in Figure 1). In this study, beef was chosen and bought from local market. Before the spiking and recovery studies, the sample was verified to not contain targets.

10 g of homogenized beef spiked with 100 *μ*L of a known concentration solution of mixed standard solution (10 *μ*g/mL, 20 *μ*g/mL, and 40 *μ*g/mL) was added to a 50 mL polypropylene centrifuge tube, and then 20 mL of 4% ammonia-methanol was added. The mixture was shaken on an orbital shaker for 5 min and then centrifuged at 12000 rpm for 20 min. The residue was extracted once more. The supernatant was collected and concentrated to dryness in a rotary evaporator at 45°C. The dry residue was redissolved in 1 mL of running buffer (35 mmol/L, pH 8.8, H_3_BO_3_-Na_2_B_4_O_7_). Then, the solution was filtered with a 0.22 *μ*m membrane filters for analysis.

### 2.4. SPE Preconcentration

The C_18_ (500 mg/6 mL) cartridge was activated with 12 mL methanol and 6 mL water to wash the impurities away. Then the fluoroquinolone was eluted with the eluent, collected into 50 mL distillation flask, and evaporated to dryness at 45°C under reduced pressure. The residue was redissolved with 1 mL of running buffer (35 mmol/L, pH 8.8, H_3_BO_3_-Na_2_B_4_O_7_). Then, the solution was filtered with a 0.22 *μ*m membrane filters for analysis.

## 3. Results and Discussion

### 3.1. Establishment of Fluoroquinolone CE Standard Method

#### 3.1.1. Wavelength

In order to measure the detection wavelength for DIF and SAR, 10 *μ*g/mL standard sample solution was scanned ranging from 190 nm to 300 nm. The maximum absorption value of the two analytes was at 275 nm, so 275 nm was selected as the detection wavelength.

#### 3.1.2. Buffer pH

**Figure 2:**
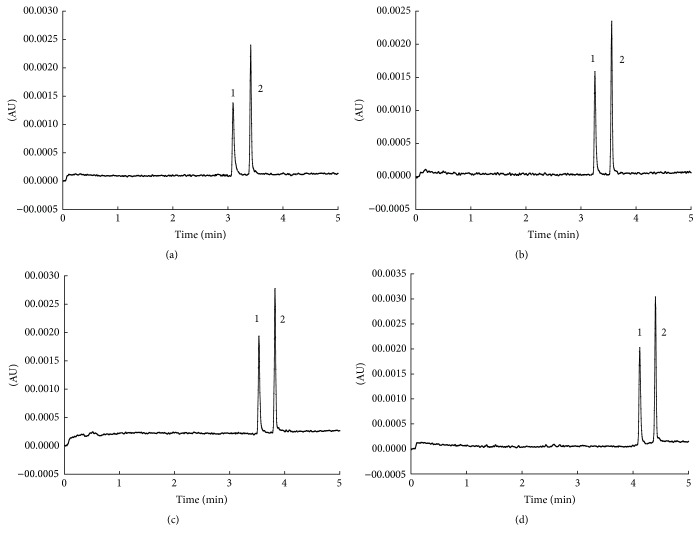
Effect of the different pH on the separation. Separation conditions: running buffer, 35 mmol/L H_3_BO_3_-Na_2_B_4_O_7_; separate voltage, 25 kV; operating temperature, 22°C; the detection wavelength, 275 nm; injection, 0.5 psi, 4 s. Chromatogram identification: (a) 8.6; (b) 8.8; (c) 9.0; (d) 9.2. Peak identification: 1: sarafloxacin; 2: difloxacin.

The pH is one of the important factors influencing the charge of targets, further affecting their migration time, so the effect of pH on the separation efficiency of SAR and DIF was investigated with pH ranging from 8.6 to 9.2. As shown in Figure 2, SAR and DIF could be baseline separated with pH increasing from 8.4 to 9.0. However, the retention time was prolonged, which might be as a result of the increase of electromigration, whose direction was opposite to electroosmotic flow. Considering the retention time, three levels of pH (pH = 8.6, 8.8, 9.0) were applied to finish the orthogonal test.

#### 3.1.3. Buffer Concentration

**Figure 3:**
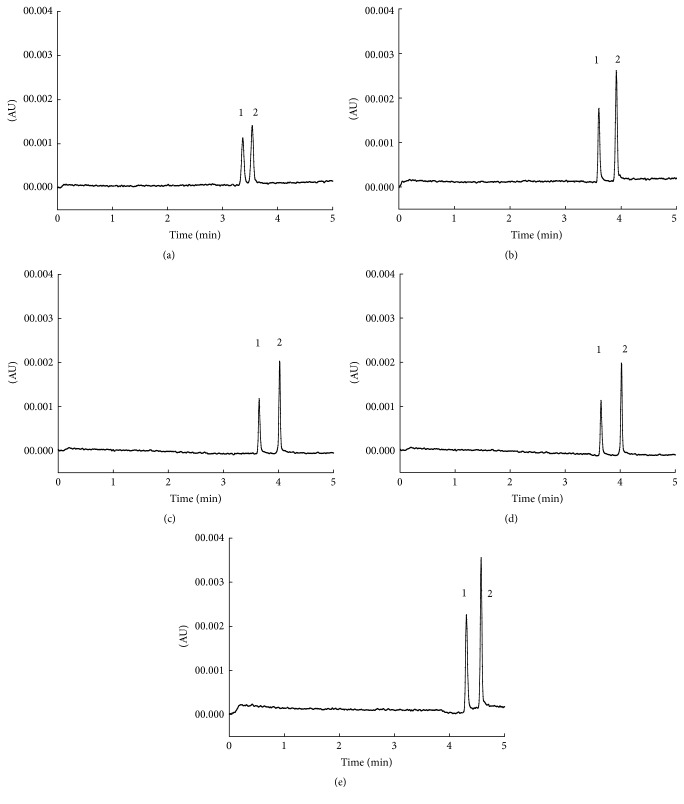
Effect of different buffer concentration on the separation. Chromatogram identification: (a) 10 mmol/L; (b) 20 mmol/L; (c) 30 mmol/L; (d) 35 mmol/L; (e) 40 mmol/L. Separation conditions and peak identification were as in Figure 2.

The influence of buffer concentrations on the separation of the SAR and DIF was also investigated from 10 to 40 mmol/L. As shown in Figure 3, the concentration of the buffer directly affected the retention time and resolution of the two analytes. When the buffer concentration was 10 mmol/L, SAR and DIF could not be baseline separated. With the buffer concentration increasing, the resolution of the two analytes improved greatly. What is more, the retention time was prolonged, which might be attributed to the fact that the higher buffer concentration resulted in the reduction of electroosmotic flow. Herein, the orthogonal test was carried out by three levels of H_3_BO_3_-Na_2_B_4_O_7_ buffer (30, 35, and 40 mmol/L).

#### 3.1.4. Separation Voltage

**Figure 4:**
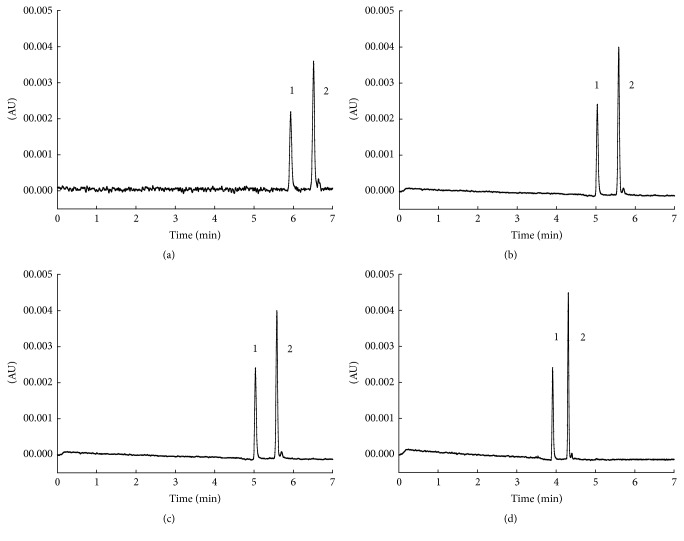
Effect of different separation voltage on the separation. Chromatogram identification: (a) 17 kV; (b) 20 kV; (c) 22 kV; (d) 25 kV. Separation conditions and peak identification were as in Figure 2.

The comparison of the effects of different separation voltages on the separation performance can be seen in Figure 4; with the separation voltage increasing, the migration time of the two analytes was shortened, along with the peak shape improving. Thus, 20, 22, and 25 kV were chosen as separation voltage to perform the orthogonal test.

#### 3.1.5. Separation Temperature

**Figure 5:**
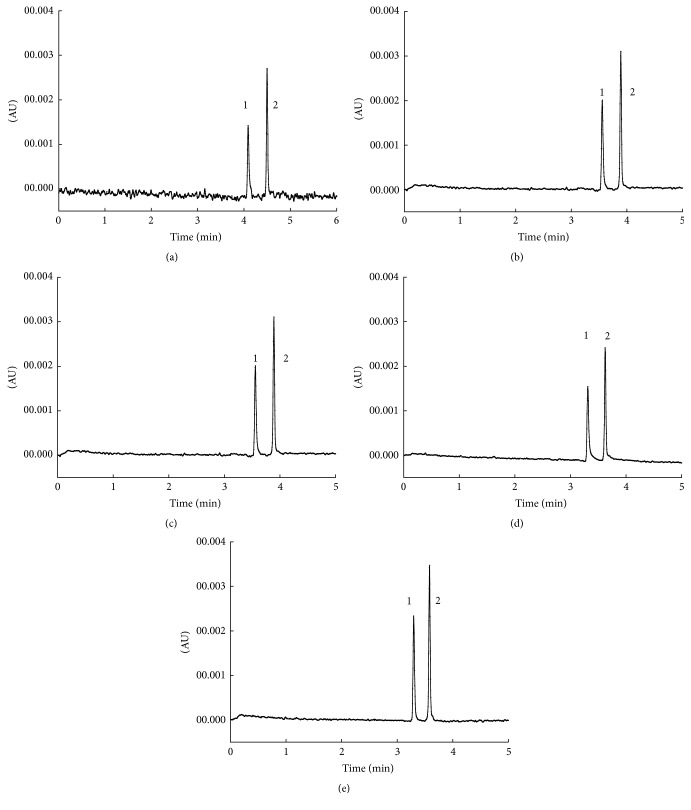
Effect of different temperature on the separation. Chromatogram identification: (a) 20°C; (b) 22°C; (c) 25°C; (d) 27°C; (e) 30°C. Separation conditions and peak identification were as in Figure 2.

The separation temperature also plays an important role in the separation efficiency and separation repeatability.  Figure 5 shows that the targets can be baseline separated with pH ranging from 20°C to 30°C, but the baseline at lower temperature is not stable. With the temperature increasing, the electroosmotic flow increased due to the decrease of viscosity, further shortening the retention time. Therefore, 22, 25, and 27°C were selected to carried out the orthogonal test.

#### 3.1.6. Orthogonal Experiment

**Figure 6:**
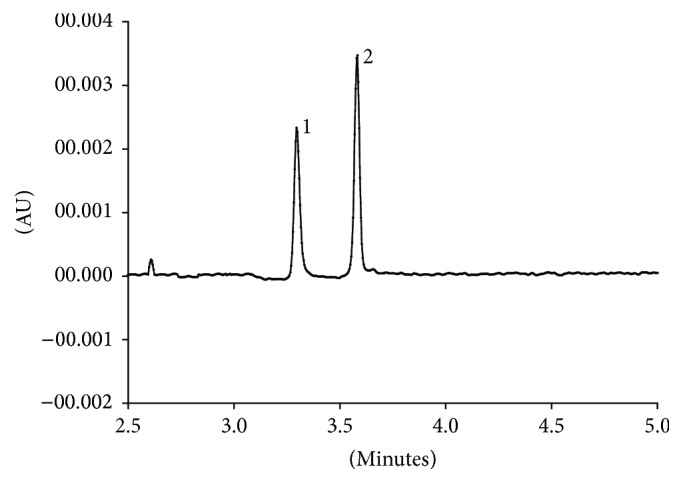
Electrophoretogram of stand solution (20 *μ*g/mL). Separation conditions: running buffer, 35 mmol/L H_3_BO_3_-Na_2_B_4_O_7_ (pH 8.8); separate voltage, 25 kV; operating temperature, 22°C; the detection wavelength, 275 nm; injection, 0.5 psi, 4 s. Peak identification was as in Figure 2.

Resolution and analysis time are key parameters in high-performance capillary electrophoresis separation, which always affect the sensitivity, analysis efficiency, and potential application of the method. Herein, in order to obtain the optimal analysis performance, separation conditions, including buffer pH and concentration and separate voltage and temperature, were systemically optimized by orthogonal experiment. The results are shown in Table 1. In view of the analysis time and resolution, the optimal capillary electrophoresis conditions were established: running buffer 35 mmol/L H_3_BO_3_-Na_2_B_4_O_7_ (pH 8.8); separate voltage: 25 kV; operating temperature: 22°C; the detection wavelength: 275 nm; injection pressure: 0.5 psi; injection time: 4 s (electrophoretogram of stand solution was shown in Figure 6).

### 3.2. Optimization of the Extraction Solution

Eluent type and concentration also played an important role in SPE preconcentration system. A favorable eluent not only should efficiently elute the target ions from the SPE column but also should not interfere with the determination of the targets. In this study, we systemically investigated the elution ability of methanol, acetonitrile, dichloromethane,* n*-hexane, and 4% ammonia-methanol (Table 2). The results showed that* n*-hexane and dichloromethane could not or partially elute the targets, which cannot meet the requirement of the experiment. When using acetonitrile and methanol as eluent, the elution efficiency was significantly improved. Particularly, the elution efficiency of 4% ammonia-methanol for the two drugs was achieved up to 98.5%–99.8%. As a result, 4% ammonia-methanol was selected as the eluent.

### 3.3. Method Evaluation

Under the optimal conditions, the analytical parameters of the current method were studied by standard solutions of the DIF and SAR, and the results were summarized in Table 3. Figure 6 showed an electrophoretogram of standard solution (20 *μ*g/mL), obtained by adopting the previous extraction procedure and capillary electrophoresis conditions. The analytical calibration curves of SAR and DIF were in the range of 1–20 *μ*g/mL (*R*^2^ = 0.9977) and 0.5–20 *μ*g/mL (*R*^2^ = 0.9936), respectively. Correlation coefficient *r* test was employed to test the linearity of the calibration curve, showing that the linearity of the proposed method was reliable at 99.9% confidence level. The limit of detection (LOD) was found to be 0.8 *μ*g/mL for SAR and 0.3 *μ*g/mL for DIF, defined as the concentration of target for which the signal-to-noise ratio was equal to three (*S*/*N* = 3). The limit of quantitation (LOQ) defined as the lower limit of linearity range for the obtained calibration curve was 1 *μ*g/mL for SAR and 0.5 *μ*g/mL for DIF. Herein, *F* test was used to test the interday differences of LOD and LOQ. The result showed that there are no significant differences at 95% confidence level. Besides, the reproducibility of the peak area for 8 *μ*g/mL SAR and DIF was tested. The RSDs for interday and intraday assay were 7.8% and 4.8%, respectively, which indicates that the current method gives a repeatable quantification of DIF and SAR.

### 3.4. Sample Analysis

To evaluate the practicability of the method, blank samples were spiked with SAR and DIF standard solution at three different concentrations (100, 200, and 400 *μ*g/kg) and then analyzed three times under the optimal conditions. Details were shown in Table 4. The recoveries were obtained in the range of 82.4–92.9% with SD (*n* = 3) lower than 6.0%. The LODs of SAR and DIF in beef were obtained at 100 *μ*g/kg and 50 *μ*g/kg, respectively. These results indicated that the current method was reliable and possessed potential application in the analysis of real samples.

### 3.5. Methodology Evaluation

To ensure human food safety, the European Union (EU) has set maximum residue limits (MRLs) of veterinary medicinal products in foodstuffs of animal origin. However, no MRLs have been defined for SAR in beef. In this study, CE-SPE which has been validated with satisfying results [2] was introduced in determination of DIF and SAR in beef. In order to obtain a better analysis performance, separation conditions were systemically optimized by orthogonal experiment. In view of the analysis time and resolution, the optimal capillary electrophoresis conditions were established. Compared to previous report [2], the detection time was shortened to 3.58 min in this paper. The LODs of SAR and DIF in beef were obtained at 100 *μ*g/kg and 50 *μ*g/kg, respectively. It is demonstrated that the proposed method allows for the rapid and sensitive detection of SAR and DIF in beef at a concentration below the MRL of EU.

## 4. Conclusion

In this work, a rapid and simple method was established allowing the determination of DIF and its metabolite SAR in beef by CE coupled with SPE. The parameters affecting the performance of developed method were systematically studied. Under the optimal conditions, good recovery, precision, sensitivity, and analysis speed were obtained. These results indicated that the proposed method might serve as a reliable tool for the effective determination of SAR and DIF in beef and other food samples.

## Figures and Tables

**Table 1:** The results of orthogonal experiment.

	Ion concentration (mmol/L)	pH	Separation voltage (kV)	Temperature (°C)	Resolution (R)	Analysis time (min)
1	30	8.6	20	22	2.1	5.38
2	30	8.8	22	25	1.8	5.02
3	30	9.0	25	27	1.7	3.46
4	35	8.6	22	27	1.0	4.82
5	35	8.8	25	22	1.9	3.58
6	35	9.0	20	25	2.8	5.70
7	40	8.6	25	25	1.8	3.52
8	40	8.8	20	27	1.1	5.28
9	40	9.0	22	22	2.0	5.17

**Table 2:** Recoveries of fluoroquinolone extracted with different solvent (%).

Eluent	SAR		DIF	
	Recovery (%)	SD	Recovery (%)	SD
Dichloromethane	54.0	0.95	58.0	0.70
Acetonitrile	85.4	0.98	82.0	1.41
Methanol	92.2	1.23	89.1	0.53
4% ammonia-methanol	98.5	1.18	99.8	1.45

**Table 3:** Analytical parameters of the current method.

Parameter	Sarafloxacin	Difloxacin
Calibration curves	*Y* = 318.50*X* − 232.95	*Y* = 517.24*X* − 85.493
Correlation coefficient (*r*^a^, *n* = 5)	0.9988	0.9967
Linearity range (*μ*g/mL)	1–20	0.5–20
LOD (*μ*g/mL) (*F*^b^, *n* = 5)	0.8 (1.60)	0.3 (3.40)
LOQ (*μ*g/mL) (*F*^b^, *n* = 5)	1 (2.12)	0.5 (4.00)

^a^Linearity test of the calibration curve. theoretical values at 99.9% confidence level: *r*_99.9%,3_ = 0.991.

^b^Interday differences test, theoretical values at 95% confidence level: *F*_95%,4_ = 6.39.

**Table 4:** Recovery study of sarafloxacin and difloxacin in spiked sample by CE (mean ± SD, *n* = 3).

Samples	Targets	Found (*µ*g/kg)	Spiked level (*µ*g/kg)	Detected (*µ*g/kg)	Recovery (%)
Beef	Sarafloxacin	0	100	91.7 ± 1.5	91.7 ± 1.5
			200	185.8 ± 6.3	92.9 ± 3.2
			400	342.7 ± 5.1	85.7 ± 1.3
	Difloxacin	0	100	90.1 ± 5.3	90.1 ± 5.3
			200	168.0 ± 4.9	84.0 ± 2.5
			400	329.4 ± 9.4	82.4 ± 2.3
